# Isothiocyanates and Glucosinolates from *Sisymbrium officinale* (L.) Scop. (“the Singers’ Plant”): Isolation and in Vitro Assays on the Somatosensory and Pain Receptor TRPA1 Channel

**DOI:** 10.3390/molecules24050949

**Published:** 2019-03-08

**Authors:** Gigliola Borgonovo, Nathan Zimbaldi, Marta Guarise, Patrizia De Nisi, Luciano De Petrocellis, Aniello Schiano Moriello, Angela Bassoli

**Affiliations:** 1 Department of Food, Environment and Nutrition-DeFENS, University of Milan, Via Celoria 2, I-20133 Milano, Italy; gigliola.borgonovo@unimi.it (G.B.); nathan.zimbaldi94@gmail.com (N.Z.); 2 Department of Agricultural and Environmental Sciences-DISAA, University of Milan, Via Celoria 2, I-20133 Milano, Italy; marta.guarise@unimi.it (M.G.); patrizia.denisi@unimi.it (P.D.N.); 3 Endocannabinoid Research Group-Institute of Biomolecular Chemistry-CNR, Pozzuoli, I-80078 Napoli, Italy; luciano.depetrocellis@icb.cnr.it (L.D.P.); aniello.schianomoriello@icb.cnr.it (A.S.M.); 4 Epitech Group SpA, Saccolongo, 35030 Padova, Italy

**Keywords:** *Sisymbrium officinale*, glucosinolates, isothiocyanates, TRPA1 ion channel, somatosensory perception

## Abstract

*Sisymbrium officinale* (L.) Scop. is a wild common plant of the Brassicaceae family. It is known as “the singers’ plant” for its traditional use in treating aphonia and vocal disability. Despite its wide use in herbal preparations, the molecular mechanism of action of *S. officinale* extracts is not known. The plant is rich in glucosinolates and isothiocyanates, which are supposed to be its active compounds. Some members of this family, in particular allylisothiocyanate, are strong agonists of the transient receptor potential ankyrin 1 (TRPA1) channel, which is involved in the somatosensory perception of pungency as well as in the nociception pathway of inflammatory pain. This study aims to isolate the glucosinolates and isothiocianates from fresh *S. officinale* to identify the major components and test their activity in in vitro assays with a cloned TRPA1 channel. Samples of cultivated *S. officinale* have been extracted and the active compounds isolated by column chromatography, HPLC and PTLC. The main components glucoputranjivin, isopropylisothiocyanate and 2-buthylisothiocianate have been tested on TRPA1. The glucosinolates glucoputranjivin and sinigrin turned out to be inactive, while isopropylisothiocyanate and 2-buthylisothiocyanate are potent agonists of TRPA1, with an EC_50_ in the range of the high potency natural agonists identified so far for this somatosensory channel.

## 1. Introduction

### 1.1. Sisymbrium officinale (Erysimum), “the Singers’ Plant”

*Sisymbrium officinale* (L.) Scop. (Brassicaceae) (SO) is an annual plant which is spread mostly in the Eurasiatic Region and North Africa. It is very common in bare ground, on roadsides, dumps and on the edges of fields. The plant is the object of a multidisciplinary project named “Erisimo a Milano” studying its diffusion and ecology in North Italy, agronomic potential, phytochemistry and food applications [1].

SO (also commonly named “erysimum” or “the singers’ plant”) is known for its traditional use for vocal tract and other upper respiratory trait diseases. The chemical markers [2] of SO are glucosinolates (GLSs) and isothiocyanates (ITCs), also commonly found in mustard oil and all brassicaceae. Historically, sulphured compounds are reputed to stimulate mucosal secretion in the upper respiratory tract, increasing expectoration [3]. The pharmacological activity of SO shows anti-inflammatory, analgesic, antitussive, myorelaxant [4] and broad-spectrum antimicrobial [5] activity and also antimutagenic properties [6]. Its effect on alleviating vocal trait disability in a cohort of 104 patients showing various degrees of vocal trait discomfort has been recently reported [7].

Despite the millennia-held beliefs [8] and recent reports regarding the plant [7], a specific mechanism of action for SO phytochemicals has not yet been described in the literature; thus, the search for the specific mechanism of action of its components is an interesting target for phytochemical research.

### 1.2. Glucosinolates and Isothiocyanates in SO

As in other plants, glucosinolates (GLSs) are present in SO with their corresponding ITCs, formed by enzymatic hydrolysis by myrosinase and/or by chemical hydrolysis that could occur during the plant manipulation, storage and processing. The formation of ITCs from GLSs via a Lossen’s type rearrangement has been extensively reviewed [9,10,11]. The relative content of GLS and ITC is often difficult to establish due to the dynamic equilibrium between the two classes of compounds and to the very different chemical features of these functional groups; GLSs are quite polar and water soluble, while ITCs are mainly low polar and volatile compounds. Analytical methodologies for their determination are therefore different; UV spectroscopy and/or HPLC are mostly used for GLSs analysis, while gas chromatography–mass spectrometry (GCMS) is more suitable for volatile ITCs.

**Figure 1:**
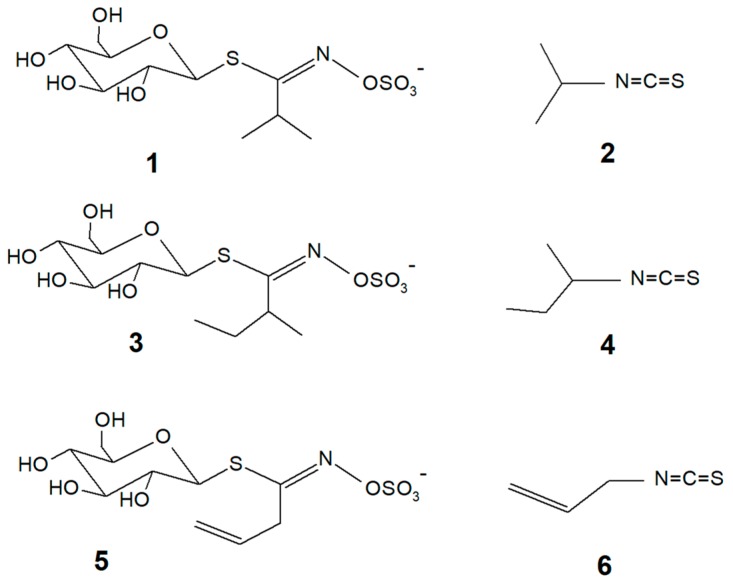
Chemical structures of compounds **1**–**6** reported in this paper.

The literature [12] identifies glucoputranjivin (compound **1**, Figure 1) as the main glucosinolate in SO; its corresponding isothiocyanate is isopropylisothiocyanate (IPITC, compound **2**).

In addition, 2-butylisothiocyanate (2-BITC, compound **4**) from glucochoclearin **3** has been previously identified in SO [5] by the GCMS of hydrodistillates. The presence of sinigrin **5** and its corresponding allylisothiocyanate (AITC) **6** is controversial; they have been identified in the fresh plant [13] but in a following report, they have been stated to be absent in SO [14]. Nevertheless, sinigrin **5** is the only commercially available standard for GLS and therefore is universally used as the reference compound for the qualitative and quantitative determination of GLSs in SO extracts [12], used also in the French pharmacopea method [15].

AITC 6 from sinigrin is the molecular marker of sinapis; it is responsible for the pungency and irritation of many food plants including mustard, wasabi and many others. AITC is also the reference compound to test the activity of chemicals on the TRPA1 somatosensory channel.

### 1.3. TRPA1 Channel as the Target of Pungent Principles in Plants

The TRPA1 channel is a protein in the family of transient receptor potential ion channels. Previously called ANKTM1 (ankyrin repeat-containing ion channel 1), TRPA1 is the only member of the TRPA sub-family reported in mammals [16]. TRPA1 is a polymodal sensor involved in the somatosensory perception of several stimuli, among which are cold, pungent compounds, airborne irritants and cannabinoids, as well as finding use in audition, proprioception, neurogenic inflammatory responses and pain perception [17,18]. TRPA1 is expressed in the dorsal root ganglion (DRG), vagal ganglion (VG), nodose and trigeminal ganglion (TG) neurons [19]. It has been reported that TRPA1 is also expressed in non-neural cells that are also relevant for chemosensation such as skin keratinocytes [20,21] and airway epithelial cells [22].

TRPA1 is activated by a large number of chemicals found in many plants, food, cosmetics, and pollutants. Electrophilic, pungent compounds such as unsaturated aldehydes, ketones, isothiocyanates, and thiosulfinates can be found in many plants, and particularly in Brassicaceae. These plants (cabbage, broccoli, cauliflower, mustard, horseradish, radish, wasabi, and many more) are important crops for human and animal diets; many of them, including SO, are also commonly used as traditional remedies. Allylisothiocyanate (mustard oil) and derivatives such as benzyl-, phenylethyl-, isopropyl-, and methyl-isothiocyanates are the main pungent ingredients inducing TRPA1 activation [23].

### 1.4. Aim of This Work

Basing on the evidence emerging from the literature that TRPA1 agonists have a role in mediating anti-inflammatory and/or analgesic effects [24,25], a possible involvement of this channel in the pharmacological mechanism of SO extracts can be envisaged. To test this hypothesis, the aim of this work is to isolate single compounds from SO and to submit them to in vitro assays with cloned TRPA1 receptor in order to verify the presence of effective agonists in the plant. Both glucosinolates and isothiocyanates were isolated and tested. Glucoputranjivin **1**, the main GLS from SO, was also synthesised in order to obtain larger amounts of the compound, to confirm its structure and to use it as a standard for analytical purposes.

## 2. Results

### 2.1. Preliminary Analysis of SO Volatiles

A preliminary analysis was performed with the aim of identifying the main volatile compounds and particularly the isothiocyanates in our samples. Volatile profiles of SO plants were obtained by solid-phase micro extraction (HS-SPME) coupled with gas chromatography–mass spectrometry (GCMS).

Hydrolysis products of glucosinolates were extracted and analyzed following literature methods [26,27].

For SPME sampling, fresh leaves were put in a vial and rapidly mashed and crushed, had water added, stoppered with a screw cap equipped with septa, and then an SPME fiber was inserted into the sample headspace to be analyzed by GCMS. These conditions were adopted to simulate the mastication process of fresh leaves, releasing myrosinase from the tissues in the oral cavity and hydrolyzing the glucosinolates to the corresponding volatile isothiocyanates. ITCs are then perceived in the oral cavity due to their pungency, mediated by the TRPA1 receptor. In our experimental conditions, the volatile ITCs were analyzed by GCMS and identified basing on their retention time, mass spectrum and by comparison with the commercially available standards.

Results show that ITCs account for the major proportion in volatiles. Hydrolysis mainly leads to IPITC (compound **2**), followed by 2-BITC (compound **4**). The percentage of the two compounds varies in different samples, with compound **2** always being the main component in leaves, seeds and flowers; compound **4** is present in variable amounts, reaching 24% of total volatiles in a sample of fresh flowers [28]. The compound 2-BITC is the hydrolysis product of glucochoclearin **3**; it is abundant in plants of the genus Cochlearia and has been previously found also in SO [5]. The sum of allylisothiocyanate, methylisothiocyanate and other isothiocyanates accounts for only about 1% of total volatiles. This result confirms that sinigrin can be present in SO as a minor component.

### 2.2. Isolation and Synthesis of Glucoputranjivin 1

For the isolation of GLSs, extracts of SO were prepared following the literature procedure [15] starting from dry plant material. A preliminary analysis showed that the seeds are more easily purified compared to leaves and flowers; therefore, the isolation of **1** was made from seeds. The extracts were purified by column chromatography, followed by PTLC. We obtained 8.5 mg of compound **1** with a purity estimated at about 94% by HPLC. The structure of glucoputranjivin **1** was confirmed by ^1^H and ^13^C-NMR spectroscopy and by comparison of spectral data to those of the desulfo-corresponding compounds described in the literature [29] and with those of the analogous glucocochlearin [30]. The NMR data obtained are shown in Table 1.

To confirm the structure of compound **1** and to obtain a larger amount, the total synthesis was performed following a literature method [31]. Compound **1**, obtained by synthesis, is chromatographically and spectroscopically identical to the natural sample from SO, and with a higher degree of purity. The availability of a pure standard sample of compound **1** facilitated the analysis and purification of extracts both by TLC and HPLC.

Despite many attempts, we could not isolate compound **3** in our samples, even if its presence is strongly suggested by the concomitant presence of its derivative ITC in the volatile fraction. Compound **3** has a 2-butyl residue in the side chain instead of the isopropyl occurring in **1**; therefore, it is likely that the two compounds have very similar chromatographic features that make the purification troublesome for compound **3**, which occurs in lower amounts in the mixture.

### 2.3. In Vitro Assays

The three pure compounds **1**, **2** and **4** were submitted to in vitro assays with the TRPA1 receptor. For comparison, sinigrin **5**, which is the reference standard for GLSs, was also tested in the same conditions. Allylisothiocyanate **6** (AITC) is conventionally used as the reference agonist for TRPA1 and was used to establish the relative efficacy for each tested compound. Antagonist/desensitizing behavior was also measured. The results of in vitro assays of isolated compounds are shown in Table 2.

**Figure 2:**
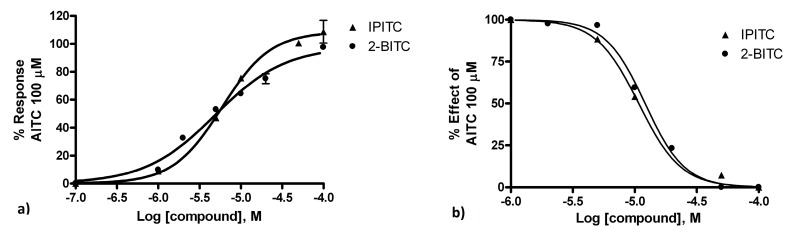
Panel (**a**): dose-related effect of isopropylisothiocyanate (IPITC) and 2-butylisothiocyanate (2-BITC) on elevation of intracellular Ca^2+^ in human embryonic kidney (HEK)-293 cells stably transfected with rat TRPA1 (rTRPA1-HEK-293). Data are expressed as percentages of the effect observed with allylisothiocyanate (AITC, 100 µM). Panel (**b**): dose-related effect of 5-min pre-incubation of rTRPA1-HEK-293 cells with IPITC and 2-BITC on the AITC (100 µM)-induced elevation of intracellular Ca^2+^. Data are expressed as percentages of the effect observed with AITC (100 µM) alone.

TRPA1-human embryonic kidney (HEK)-293 cells exhibited a sharp increase in intracellular Ca^2+^ upon application of the compounds examined—that is, **2** and **4**—with efficacies of 108.8 and 98.0, respectively, and EC_50_ values < 6 μM (Figure 2, panel a). Both **2** and **4** at 100 μM showed no activity (no elevation of internal Ca^2+^) on not-transfected HEK cells (with an activity less than 1%).

Five minutes of preincubation of TRPA1-HEK-293 cells with ITCs **2** and **4** followed, and then incubation was continued with AITC, causing the inhibition of TRPA1 response to this agonist with IC_50_ values of 10.8 and 12.1 μM, respectively (Figure 2, panel b). Then, upon activation, calcium enters the cell and stimulates a series of calcium-dependent processes that ultimately lead to the desensitization of the TRPA1 channel. Upon desensitization, the TRPA1 channel enters a refractory period in which it can no longer respond to agonist (AITC) stimulation. The EC_50_ and IC_50_ values were comparable, of the same order of magnitude. This implies that ITCs **2** and **4** desensitize the receptor to the next AITC action leading to a paradoxical inhibitory effect of these compounds.

## 3. Discussion

The use of SO extracts for voice care is widespread. Singers, actors and other professional users of the voice use commercial products containing SO (often in combination with other botanicals) for this purpose, and recently clinical studies [7] confirmed its efficacy in the treatment of vocal discomfort.

Despite the long history of use and the phytochemical knowledge of Sisymbrium, its mechanism of action on the voice is still unclear. The effect on pharyngeal irritation is suggested to be related to the content of mucilage (10.9–13.5%) [7], and Di Sotto [14] highlighted that the extract of *Sisymbrium officinale* possesses a myorelaxant activity, particularly against the agents involved in the inflammatory process. Nevertheless, a specific biological target for its active principles has never been identified.

Several literature reports confirm that the TRPA1 ion channel is a polymodal sensor involved in mediating inflammatory pain signals. Plenty of plants containing TRPA1 agonists are used for their anti-inflammatory and/or anti-nociceptive features, among which are turmeric, cinnamon, clove, ginger and many more.

Plants in the Brassicaceae family are rich in TRPA1 active compounds, and some of them are already recognized as potent agonists, as with the AITC contained in mustard. Also, other isothiocyanate compounds, including benzyl-, phenylethyl-, isopropyl- and methylisothiocyanate, have been previously shown to activate the human TRPA1 channel by calcium imaging, but the activity was expressed only as a range [23,25,32].

Our results indicate that both the ITCs contained in SO are potent agonists of the TRPA1 ion channel. In vitro, the efficacy of IPITC (compound **2**) is even higher than that of AITC, the reference standard compound for this chemical class. Compound **4**, 2-BITC, has been tested for the first time and its efficacy turned out to be high as well, although a little lower than that of AITC.

**Figure 3:**
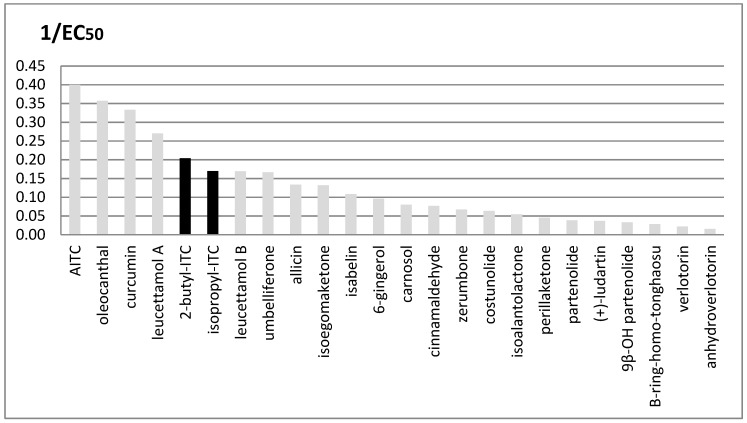
Comparison between TRPA1’s activity of ITC from *Sisymbrium officinale* (L.) Scop. and other natural known agonists. Potency is expressed as 1/EC_50_; EC_50_ values are given in microM units. Compounds described in this paper are shown in black.

The potency values of the two ITCs from SO have been compared to that of many natural agonists of the TRPA1 ion channel owing to their different chemical families, which have been recently reported by some researchers [33] (Figure 3).

The two ITCs that are present in SO extracts are among the highest-potency agonists, which include oleocanthal, curcumin and umbelliferone, whereas other well-known agonists such as cynnamaldehyde and 6-gingerol are less potent.

The involvement of sulphur derivatives in the therapeutic action of SO, as well as of many other medicinal plants in the Brassicaceae family, is usually expressed as a matter of fact. The total amount of GLSs in the plant and in commercial phytopreparations (usually expressed as sinigrin equivalents) is measured in French Pharmacopea as an index of the “goodness” of phytopreparates; this implies that GLSs are considered to be the active principles. Accordingly, traditional use suggests that the more efficient parts of the plant are the top edges and flowers, where the amount of GLSs is higher. Nevertheless, to our knowledge, there is no direct evidence that these sulphur compounds could exert a specific pharmacological activity on vocal traits.

Our data support the hypothesis that ITC compounds **2** and **4** could be among the active principles in SO, indicating the TRPA1 ion channel as the molecular target of their pharmacological action in vitro.

In our assays on the TRPA1 channel, the glucosinolates sinigrin **5** and glucoputranjivin **1** were shown to be ineffective *per se*. Since the active ITCs are produced in situ by GLS hydrolysis, this finding supports the idea that the efficacy of plant preparation is positively associated with high GLC content in the plant, which corresponds to a high content in ITCs. Furthermore, this result indicates that the method used for processing and assuming the plant material could be relevant in determining the final content in active phytochemicals and therefore the efficacy of therapeutic action.

The availability and use of pure glucoputranjivin **1** as a reference compound (instead of sinigrin) is very useful for qualitative and quantitative determinations of active compounds in SO; in fact, compound **1** shows the most abundant GLS in all parts of the plant, whereas sinigrin is usually absent or present only in traces.

The potential of active compounds from SO for applications in voice care represents an interesting topic for further studies. Despite the fact that professional vocalists are a numerically small population and that vocal problems are usually minor pathologies, the protection of the voice and the maintenance of its performance are still important targets for artists and therefore have a high intrinsic value.

The market demand for natural remedies is rapidly growing, but very often research data demonstrating the real pharmacological activity of single molecules are lacking.

The involvement of TRPA1 in one of the possible mechanisms involved in the effects on voice care of SO extracts must of course be confirmed by further in vivo and clinical experiments. The identification of active ITCs from “the singers’ plant”, their molecular targeting and in vitro measurable effects are results which can contribute to clarifying its real potential and to addressing its uses and applications.

## 4. Materials and Methods

### 4.1. Plant Material

*Sisymbrium officinale* was cultivated at the Faculty of Food and Agricultural Sciences at the University of Milano [34]. Plants were harvested during the flowering season (May–July 2017 and 2018) and seeds collected in August, September and October 2017 and 2018. The aerial parts (leaves, flowers) of the plant were dried at room temperature and stored in paper bags. Seeds were stored in a vacuum at 4 °C. For the GCMS headspace analysis of volatiles, samples were harvested from the living plant and used immediately.

### 4.2. Chemicals and Equipment

Sinigrin monohydrate (potassium salt) (CAS 3952-98-5), isopropylisothyocianate (2253-73-8), 2-butylisothiocianate (4426-79-3), methanol and acetonitrile were from Sigma-Aldrich (Milan, Italy).

Thin-layer chromatography (TLC) was performed on silica gel 60 F254 plates (Sigma-Aldrich, Italy) and developed using n-butanol/acetic acid/water 4:1:4 (*v*/*v*/*v*) as a mobile phase. The spots were visualized under UV light at 254 nm and then sprayed with a solution of thymol 1% and sulphuric acid 10% in ethanol. Sinigrin was used as a reference for GLSs; it gave a pink spot with Rf 0,2 after heating, while glucoputranjivine gave a blue–violet-colored spot (Rf 0,18).

NMR spectra were recorded on a Bruker Avance spectrometer (Bruker BioSpin GmbH, Rheinstetten, Germany) operating at 600 MHz, using MeOH-d4 as a solvent and TMS as an internal standard; J values are given in Hertz.

HPLC analysis were performed with a liquid Chromatograph Dynamax SD200 (VARIAN^®,^ Woburn, MA, US), equipped with a binary pump with Rheodyne injector and a UV-VIS detector managed by the Galaxy chemstation (VARIAN^®^, Woburn, MA, US). A reversed phase column C18 Lichrosphere (250 mm length, 4.6 mm ID, 5 μ, Phenomenex^®^) was used. The samples were dissolved in methanol and filtered with 0.45 μm nylon filters. The conditions used were the following: flow 0.7 mL/min, λ 227 nm; solution A ammonium acetate 0.01 M; solution B acetonitrile; gradient elution: t 0–10min: 100% A; t 10–15 min: A 95%, B 5%; t 15–25 min: A 95%, B 5%; t 25–45 min: A 30%, B70%.

### 4.3. HS- SPME Coupled with GCMS Analysis

Autolyzed samples were prepared by crushing 0.5 g of flowering sample plants with 1 mL of H_2_O in a 20 mL vial (Agilent Technologies, Santa Clara, CA, US), which was rapidly sealed with a magnetic screw cap equipped with silicon/polytetrafluoroethylene septa (PTFE).

Headspace (HS) volatile compounds were collected using a SPME fiber (Supelco, Darmstad, Germany) 2 cm × 50/30 m thickness coated with DVB/CAR/PDMS (Divinylbenzene/Carboxen/Polydimethylsiloxane). Initially, vials were tempered at 37 °C for 2 min. Then, the volatiles were extracted by exposing the fiber to the vial headspace for 10 min under continuous agitation and heating at 37 °C. The extracted volatiles were desorbed in the GC injection port for 2 min at 230 °C in a splitless mode. The incubation of the vials, extraction, and desorption of the volatiles were performed automatically by a CombiPAL autosampler (CTC Analytics, Zwingen, Switzerland). Chromatography was performed on a DB-5 MS (30 m × 0.25 mm × 0.25 μm) column (Agilent J&W Capillary columns, Santa Clara, CA, US) with helium as carrier gas at a constant flow of 1.0 mL/min. The GC interface, MS source and quad temperatures were 260 °C, 230 °C and 150° C, respectively. The oven temperature conditions were 40 °C for 3 min, a 5 °C/min ramp until 250 °C, and then held at 250 °C for 5 min. Mass spectra were recorded in scan mode in the 30 to 300 mass-to-charge ratio range by a 5975B mass spectrometer (Agilent Technologies) at an ionization energy of 70 eV and a scanning speed of 7 scans/s. Chromatograms and spectra were recorded and processed using the Enhanced ChemStation software (Agilent Technologies). GCMS total ion chromatogram (TIC) and mass spectra are available as Supplementary Materials (S1, S2).

### 4.4. Glucoputranjivin, Isolation and Purification

Glucoputranjivin **1** was obtained from seeds. 10.3 grams of seeds were extracted with 200 mL of 80% MeOH at 80 °C for 30 min. After the first extraction, the seeds were shredded in a mortar and extracted again with hot MeOH (160 mL for 30 min). After the evaporation of the solvent in a vacuum, 992.5 mg of crude extract were obtained. The crude extract was purified by two subsequent flash column chromatographies (DCM/MeOH 60:40). From the second column, only one fraction (43.5 mg), the richest in glucoputranjivin, was submitted to purification by PTLC (DCM/MeOH 65:35) to give compound **1** (8.5 mg) with a purity >90% by HPLC and NMR. Compound **1** (19 mg) was synthesized by the literature method [31] (Supplementary Material, Figure S3); the purity was >90% (HPLC, Figure S5). Intermediate compounds in the synthesis have already been described in the literature [31,35]; experimental analytical data are available in Supplementary Materials (S4).

Compound **1** is an amorphous solid; *m*/*z*: HRMS *m*/*z* (C10H18NO9S2): 360.0421 (100%, M+), 361.0450 (12%, M + 1), 362.0395 (10%, M + 2), 336.3266 (30%); calculated mass: 360.0423. IR (ν, cm^−1^) 1572.18 (-C=N-), 1272.29 (-O-SO_3_^−^). ^1^H and ^13^C NMR data are reported in Table 1; full spectra are reported in Supplementary Material S6–S9.

### 4.5. In Vitro Assays with rTRP Ion Channels

Compound effects on intracellular Ca^2+^ concentration ([Ca^2+^]_i_) were determined using the selective intracellular fluorescent probe Fluo-4. Assays of the rat TRPA1-mediated elevation of intracellular Ca^2+^ in transfected HEK-293 cells were performed as described [36]. Briefly, human embryonic kidney (HEK-293) cells (stably transfected with rat TRPA1 or not transfected) were cultured in EMEM + 2 mM Glutamine + 1 % non-essential amino acids (NEAA) + 10 % FBS and maintained at 37 °C with 5 % CO_2_. On the day of the experiment, the cells were loaded with Fluo-4 AM (4 μM) for 1 h at room temperature, rinsed and resuspended in Tyrode’s solution (145 mM NaCl, 2.5 mM KCl, 1.5 mM CaCl_2_, 1.2 mM MgCl_2_, 10 mM d-glucose, and 10 mM HEPES, pH 7.4), and finally transferred to a quartz cuvette of a spectrofluorimeter (Perkin-Elmer LS50B, Perkin Elmer Inc, Buckinghamshire, UK); λ_EX_ = 488 nm, λ_EM_ = 516 nm) equipped with a PTP-1 Fluorescence Peltier System (PerkinElmer Life and Analytical Sciences, Waltham, MA, USA) under continuous stirring. Cell fluorescence before and after the addition of various concentrations of test compounds was measured, normalizing the effects against the response to ionomycin (4 µM). The values of the effect on [Ca^2+^]_i_ in not-transfected HEK-293 cells were used as a baseline and subtracted from the values obtained from transfected cells. Agonist efficacy was expressed as a percentage of the effect on [Ca^2+^]_i_ observed with 100 μM allylisothiocyanate (AITC). The potency of the compounds (EC_50_ values) was determined as the concentration required to produce half-maximal increases in [Ca^2+^]_i_. Antagonist/desensitizing behavior as evaluated against TRPA1 agonist AITC 100 μM by adding the compound directly into the quartz cuvette 5 min before the stimulation of cells with the agonist. IC_50_ is expressed as the concentration exerting a half-maximal inhibition of an agonist effect, taking as 100% the effect on [Ca^2+^]_i_ exerted by the agonist alone. Dose–response curves are fitted by a sigmoidal regression with a variable slope. Curve fitting and parameter estimation were performed with a GraphPad Prism (GraphPad Software Inc., San Diego, CA). Three independent experiments were done for each determination; all determinations were performed at least in triplicate. The time-course spectra showing the increase of intracellular Ca^2+^ upon application of the compounds are available in Supplementary Material (S10).

## Figures and Tables

**Table 1:** ^1^H and ^13^C data for glucoputranjivin **1** in MeOH-d4 at 25 °C.

C	Assignment	δ_C_ (ppm)	δ_H_ (ppm, Molteplicity, (J in Hz))
Aliphatic moiety			
1	C	165.68	
2	CH	34.03	2.97, m (6.90)
3	CH_3_	21.33 *	1.298 *, d (6.90)
4	CH_3_	22.45 *	1.296 *, d (6.90)
Glucose moiety			
1’	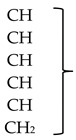		4.89, d (9.75)
2’			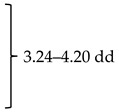
3’		62.78–86.91	
4’			
5’			
6’			

* Chemical shift after resolution enhancement; these attributions can be interchanged.

**Table 2:** In vitro assays of isolated compounds on transient receptor potential ankyrin 1 (TRPA1) receptor.

		TRPA1		
cpd	Name	Efficacy (%AITC 100μM)	Potency EC_50_ μM	IC_50_ Inhibition TRPA1 μM (AITC 100μM)
**5**	(−)-Sinigrin	<10	NA	>100
**1**	Gluco-putranjivine	<10	NA	>100
**2**	Isopropyl isothiocyanate	108.8 ± 1.6	5.9 ± 0.4	10.8 ± 0.4
**4**	2-buthyl isothiocyanate	98.0 ± 1.0	4.9 ± 0.2	12.1 ± 0.2

## Supplementary Materials

The following are available online. Figure S1: GCMS Total Ion Chromatogram (TIC) of volatiles in SO. Figure S2: Mass spectra of volatiles in SO. Figure S3: synthetic scheme for the synthesis of glucoputranjivine. Figure S4: analytical data for intermediates in the synthesis of glucoputranjivin. Figure S5: HPLC chromatogram of compound **1**. Figure S6: ^1^HNMR spectra of compound **1.** Figure S7: ^13^CNMR spectra of compound **1.** Figure S8: HRMS spectra of compound **1.** Figure S9: IR spectrum of compound **1.** Figure S10: Representative traces of [Ca^2+^]_i_ increase evoked by IPITC (a), 2-BITC (b) and AITC (c, for comparison) at 100 μM in HEK293 cells over-expressing rat TRPA1.
