# Supplementary material for: Isothiocyanates and Glucosinolates from Sisymbrium officinale (L.) Scop. (“the Singers’ Plant”): Isolation and in Vitro Assays on the Somatosensory and Pain Receptor TRPA1 Channel
Source: Molecules. 2019 Mar 8;24(5):949. doi: 10.3390/molecules24050949 (PMC6429275; doi:10.3390/molecules24050949)
Supplement: Supplementary file 1 [file molecules-24-00949-s001.pdf]

Supplementary material S1

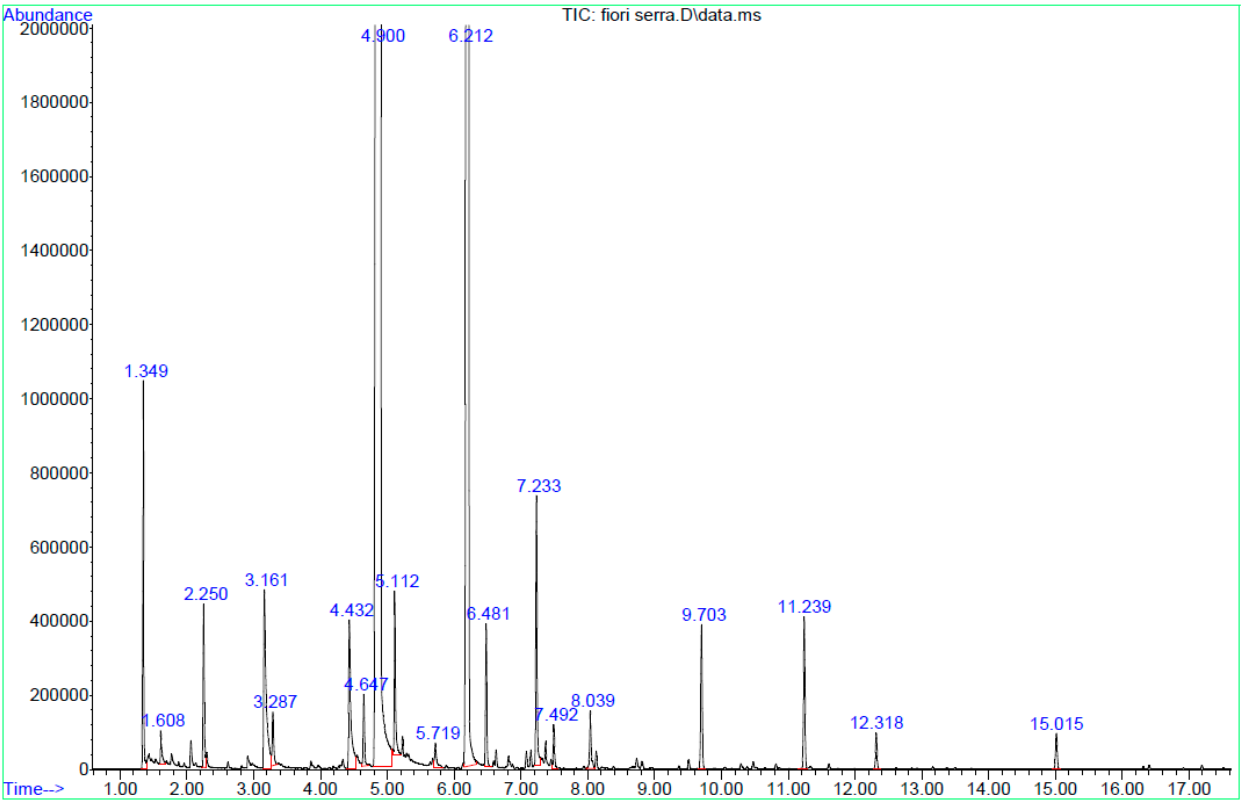

Figure S1. GC-MS Total Ion Chromatogram (TIC) of Sisymbrium officinale volatiles extracted by HS-SPME and mass spectra data of IPTC (peak 1) and 2-BITC (peak 2). Compounds were identified by mass spectra comparison with NIST08 Mass Spec. Library, RT Retention Time and comparison with authentic samples.

N. Peak		MS spectra data m/z	R_T (min)
1	IPITC	101(M^+), 86, 60, 43, 41	4,90
2	2-BITC	115(M^+), 86, 56, 41	6,21

a)

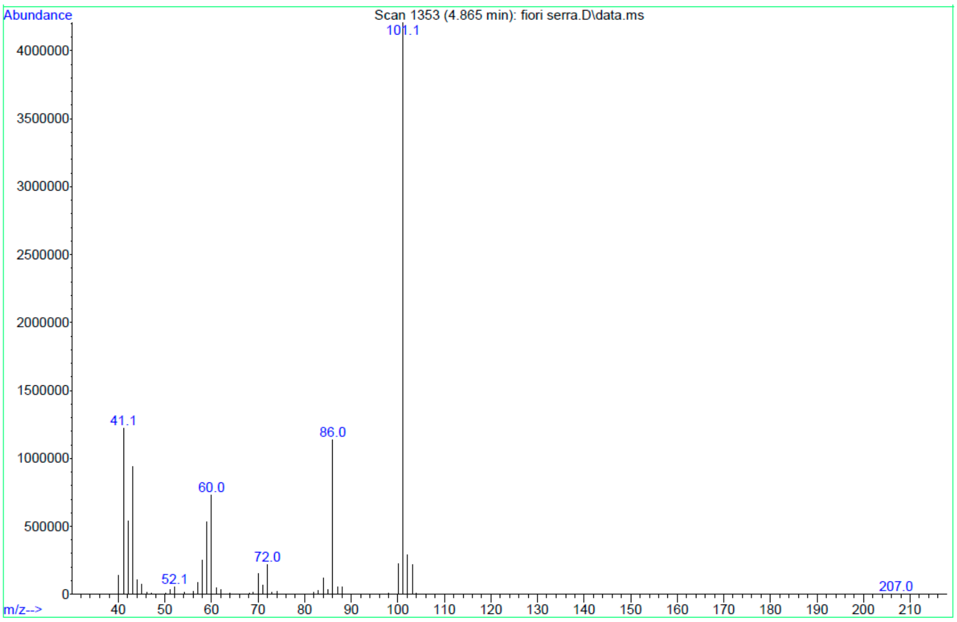

b)

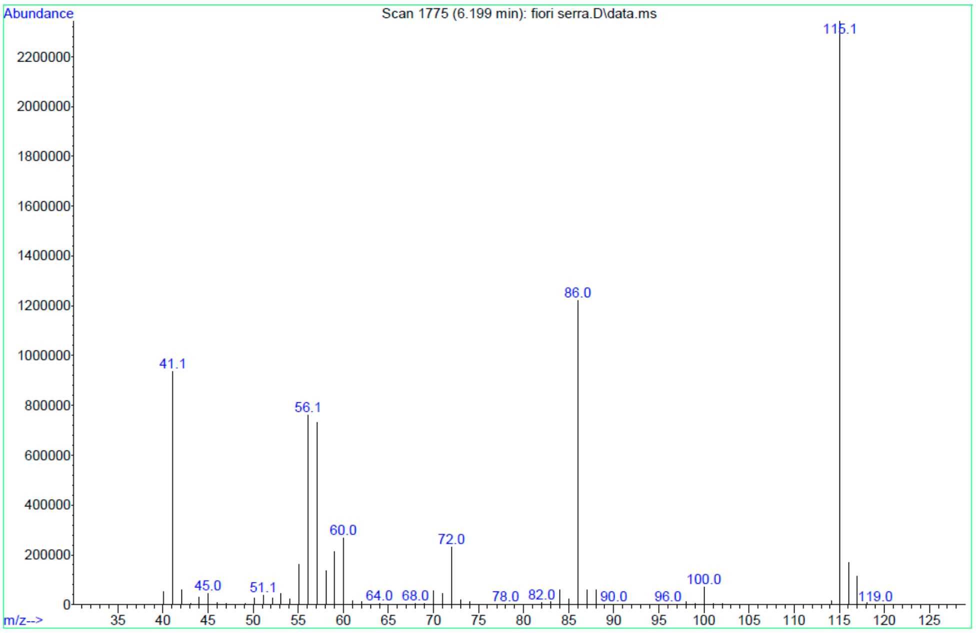

Figure S2. Mass spectra of IPTC (a) and 2-BITC (b), correspond respectively to peak 1 and peak 2 in Figure S1.

Figure S3: Synthetic scheme for the synthesis of glucoputranjivin, compound 1.

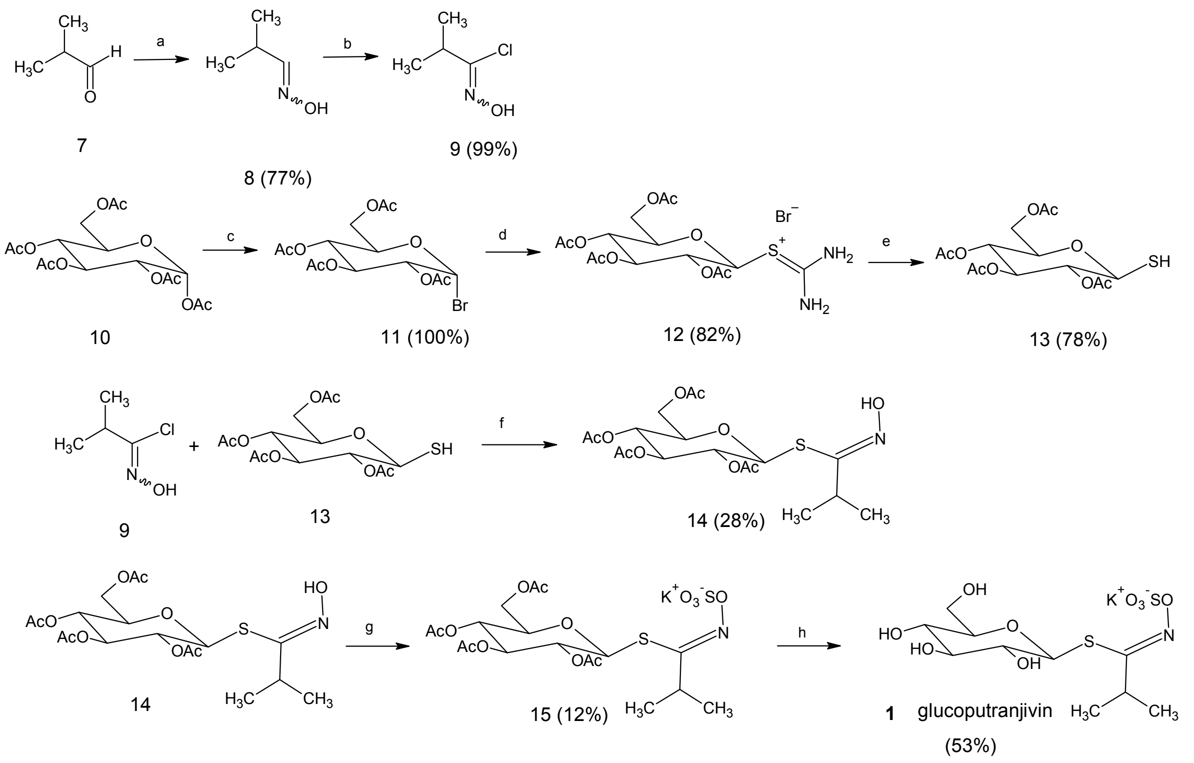

a: NH_2OH HCl, Na_2CO_3,H_2O, reflux; b: NCS; c: HBr, AcOH; d: thiourea, py; e: $Na_2S_2O_5$, DCM,H_2O; f: THF, Et_3N, g: $ClSO_3H$, py, DCM; h: KOMe, MeOH.

Supplementary material S4: Synthesis of compound **1**.

For the synthesis of compound **1** we follow the already published synthetic scheme (reference [31]: Davidson N. E.; Rutherford T. J.; Botting N.P. Synthesis, analysis and rearrangement of novel unnatural glucosinolates. *Carbohydr. Research* **2001**, 330, 295–307, DOI 10.1016/S0008-6215(00)00308-6.)

Compounds 7 and 10 are commercially available (Aldrich). Intermediates 8, 9, 11, 12 and 15 were characterized by ^{1}H-NMR as crude compounds and were used without further purification.

Compound 8: yellow oil, 1.77 g, 29.5%. ^{1}H-NMR (600 MHz, $CDCl_3$) δ (ppm): 9.42 (1 H, br s, NOH), 7.36 (1 H, d, J=5.94 Hz, CH=N), 2.57 (1 H, m, CH), 1.18, 1.08 (2 H, d, J=7.00, 2xCH3).

Compound 9: white solid (1.98 g, 80%); crystallized from dichloromethane to give 0.37 g. ^{1}H-NMR (600 MHz, $CDCl_3$) δ (ppm): 9.38 (1 H, br s, NOH), 2.50 (1 H, m, CH), 1.26, 1.20 (2 H, d, J=6.99, 2xCH3).

Compound 11: white solid (5.24 g, 99%). ^{1}H-NMR (600 MHz, $CDCl_3$) δ (ppm): 6.60 (d, 1 H, H-1, J = 4.1 Hz), 5.55 (at, 1 H, H-3), 5.15 (at, 1 H, H-4), 4.83 (dd, 1 H, H-2, J = 9.46 Hz), 4.30 (m, 2 H, H-6, H-5), 4.12 (m, 1 H, H-6′), 2.10, 2.09, 2.04, 2.03 (4s, 4 × OC(O)CH3).

Compound 12: white powder (1.97 g, 31.7%). ^{1}H-NMR (600 MHz, d6-DMSO) δ (ppm): 9.21 (br s, 4 H, 2 × NH2), 5.74 (d, 1H, H-1, J = 10.0 Hz), 5.28 (at, 1 H, H-3), 5.08 (m, 2 H, H-4, H-2), 4.12 (m, 2 H, H-5, H-6, H-7), 2.03, 2.00, 1.97, 1.95 (4s, 4 × OC(O)CH3).

<u>Compound 13</u>: 2,3,4,6-tetra-O-acetyl-1-thio-β-D-glucopyranose. White solid (1.05 g, 63.6%). ^{1}H-NMR (600 MHz, $CDCl_3$) δ (ppm): 5.19 (at, 1 H, H-3), 5.10 (at, 1 H, H-4), 4.97 (at, 1 H, H-2), 4.54 (at, 1 H, H-1), 4.25 (dd, 1 H, H-6, J = 12.37 Hz, J = 4.67 Hz), 4.13 (d, 1 H, H-6′, J = 12,37 Hz), 3.72 (ddd, 1 H, H-5, J = 9.8 Hz), 2.30 (d, 1 H, SH, J = 9.90 Hz), 2.09, 2.08, 2.02, 2.00 (4s, 4 × OC(O)CH3). ^{13}C NMR (75 MHz, $CDCl_3$) δ (ppm): 170.64, 170.11, 169.62, 169.35 (4 × OC(O)CH3), 78.73 (C-1), 77.21 (C-5), 76.79, 76.36 (C-2/C-3), 68.12 (C-4), 62.00 (C-6), 20.89, 20.71, 20.67, 20.55 (4 × OC(O)CH3).

<u>Compound 14</u>: 2,3,4,6-Tetra-O-acetyl-β-D-glucopyranosyl-1-isopropyl thiohydroximate.
Compound 13 (1.46 g, 3.91 mmol) in dry tetrahydrofurane (30 ml) was added of crude compound 9 (0.47 g, 3.91 mmol) dissolved in dry tetrahydrofurane (30 ml). Dry triethylamine (3.71 g, 36.7 mmol) was added and the mixture stirred under nitrogen at room temperature for 27 h. Diethyl ether was added and the solution washed with 1M H_2SO_4 to pH 6.0. The organic phase was concentrated in vacuo and extracted with ethyl acetate. The organic layer was dried and evaporated in vacuo. The product was purified by flash chromatography using as eluent a mixture of hexane/ethyl acetate 1:1 vol/vol; Rf of compound 14 in TLC with the same eluent is 0.33. Compound 14 was obtained as a white solid, 0.49 g, yield 28%. Mp 99°C. HRMS m/z ($C_{18}H_{27}NO_{10}S$): 472.1253 (35%, M+Na). ^{1}H NMR (CDCl3) δ: 5.24 (1H, d, J 9.48 Hz, H-1), 4.1-5.2 (6H, m), 2.76 (1H, m, CH), 1.98,2.00,2.01, 2.03 (12H, 4 s, 4 CH3COO), 1.21 and 1.22 (6H, 2 d, J 6.80 Hz, 2 CH3). ^{13}C NMR (CDCl3) δ: 170.52, 170.12, 169.31, 169.19, 157.31 (CN), 80.36, 75.89, 73.75, 70.00, 68.80, 62.12, 33.38, 20.57, 20.47, 18.98, 14.13.

<u>Compound 15</u>: 2,3,4,6-Tetra-O-acetyl-1-isopropyl glucosinolate.
A solution of chlorosulphonic acid (1.12 g, 9.60 mmol) in dry dichloromethane (10 ml) was added in 30 minutes to a stirred solution of pyridine (1.44 g, 18.24 mmol) in dry dichloromethane (10 ml) at 0°C under nitrogen atmosphere. Compound 14 (0.44 g, 0.96 mmol) in dry dichloromethane (8 ml) was added. The mixture was stirred at rt for 23 h. Potassium hydrogen carbonate (0.6 g, 4.72 mmol) in 36 ml water was added and the solution stirred for 30 min. Organic layer evaporated and a further aq potassium hydrogen carbonate (0.6g, 4.72 mmol) in water was added and the solution extracted with ethyl acetate. Purification by column chromatography on silica gel (eluent hexane/ethyl acetate 6:4) allowed recovery of 64 mg of compound 15 as white solid (yield 12%). ^{1}H NMR (aceton-d6) δ: 5.49 (1H, d, J 10.20 Hz, H-1), 4.1-5.3 (6H, m), 3.11 (1H, m, CH), 1.17 and 1.30 (6H, 2d, J 6.57 Hz, 2 CH3). ^{13}C NMR (aceton-d6) δ: 171.14, 170.87, 170.62, 168.70, 168.70 (CN), 81.21, 77.38, 74.96, 70.97, 69.96, 63.95, 34.32, 23.21, 23.11, 22.23, 21.50, 21.44, 21.38.

<u>Compound **1**</u>: glucoputranjivin.
A catalytic amount of potassium methoxide was added to a solution of compound 15 (0.05 g, 0.089 mmol) in dry methanol (5 ml) at room temperature under nitrogen atmosphere. The resulting solution was stirred overnight. Concentration under reduced pressure afforded **1** as a yellowish solid (0.019 g, 53%).
HRMS m/z ($C_{10}H_{18}NO_9S_2$): 360.0421 (100%, M+), 361.0450 (12%, M+1), 362.0395 (10%, M+2), 336.3266 (30%). Calculated mass: 360.0423. IR: 1572.18 (-C=N-), 1272.29 (-O-SO_3^-). ^{1}H and ^{13}C NMR data are reported in Table 1. Spectra are shown in comparison with those calculated by the simulation tool in ChemOffice.

Figure S5: HPLC analysis of compound **1.** RPC18 Lichrosphere (250 mm length, 4.6 mm ID, 5 μ, Phenomenex®). Flow 0.7 mL/min, λ 227nm; solution A ammonium acetate 0.01M; solution B acetonitrile. Gradient elution: t 0-10min: 100% A; t 10-15 min: A 95%, B 5%; t 15-25 min: A 95%, B 5%; t 25-45 min: A 30%, B70%.

Chromatogram : NZ ER_extr8 1in_channel1

System : Hplc
Method : gigliola
User : User1
55,00

Acquired : 21/02/2019 16.18.56
Processed : 01/01/2002 0.23.26
Printed : 21/02/2019 16.18.56

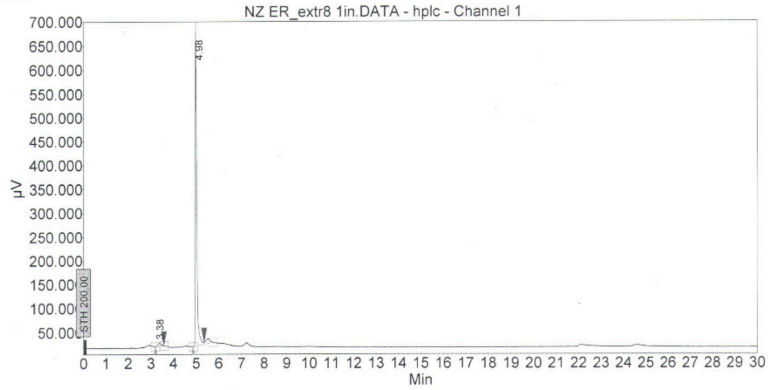

Peak results :

Index	Name	Time [Min]	Quantity [% Area]	Height [μV]	Area [μV.Min]	Area % [%]
1	3.38	3,38	2,01	6287,7	1053,2	2,012
2	4.98	4,98	97,99	683181,0	51281,0	97,988
Total			100,00	689468,7	52334,2	100,000

Figure S6: ^{1}H NMR:of compound **1**; a) experimental b) simulated

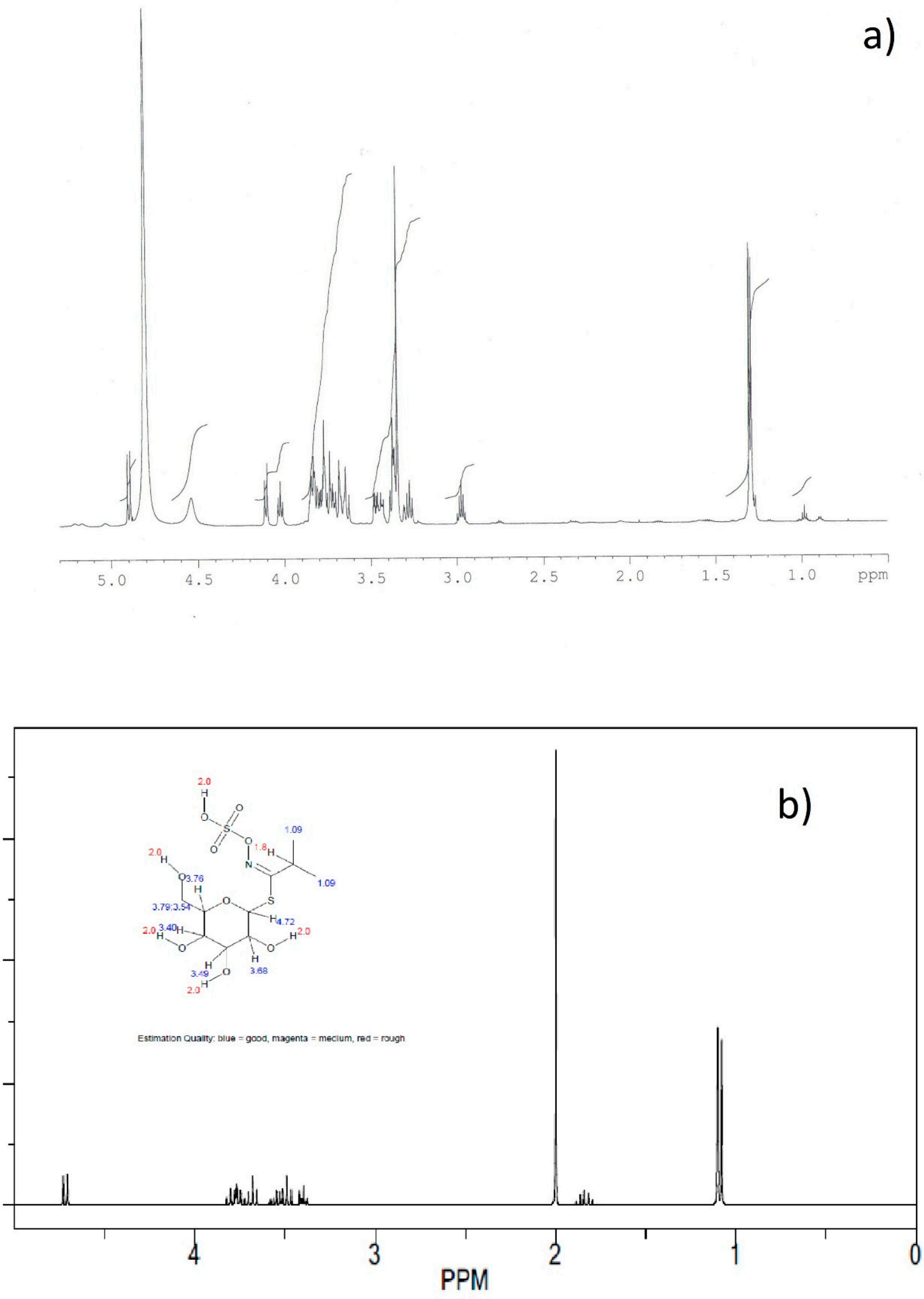

Figure S7: ^{13}C NMR:of compound **1**; a) experimental b) simulated

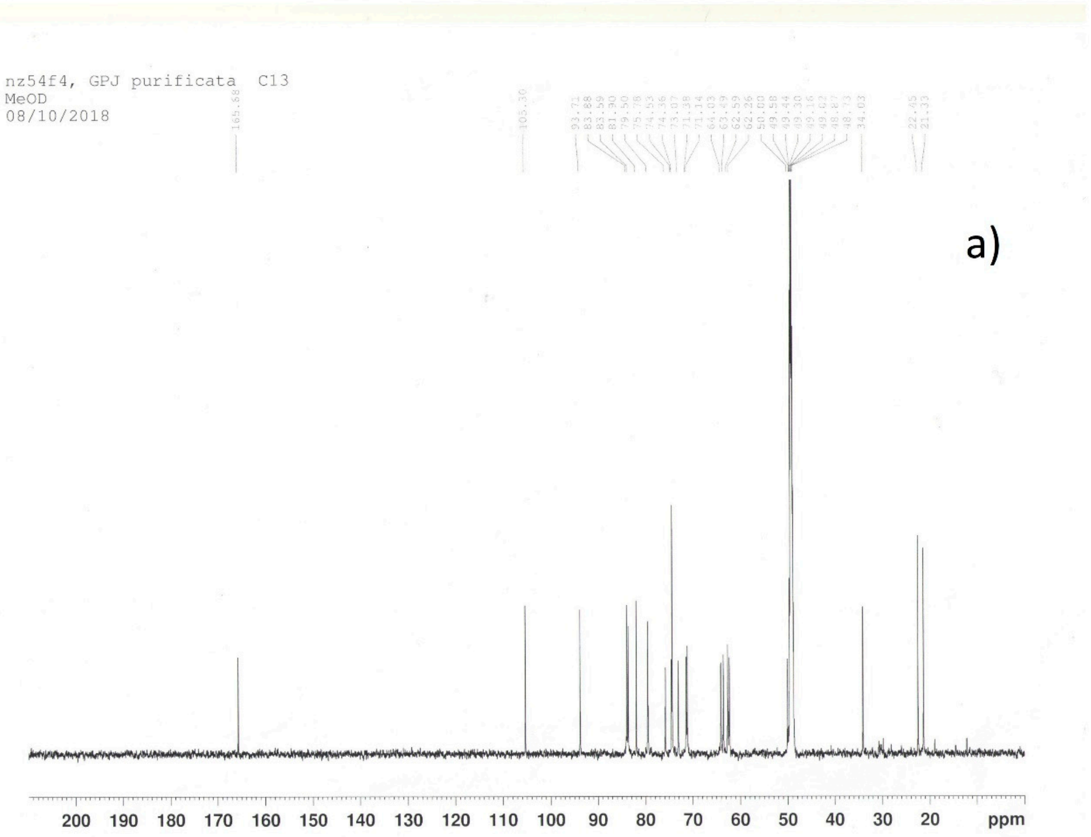

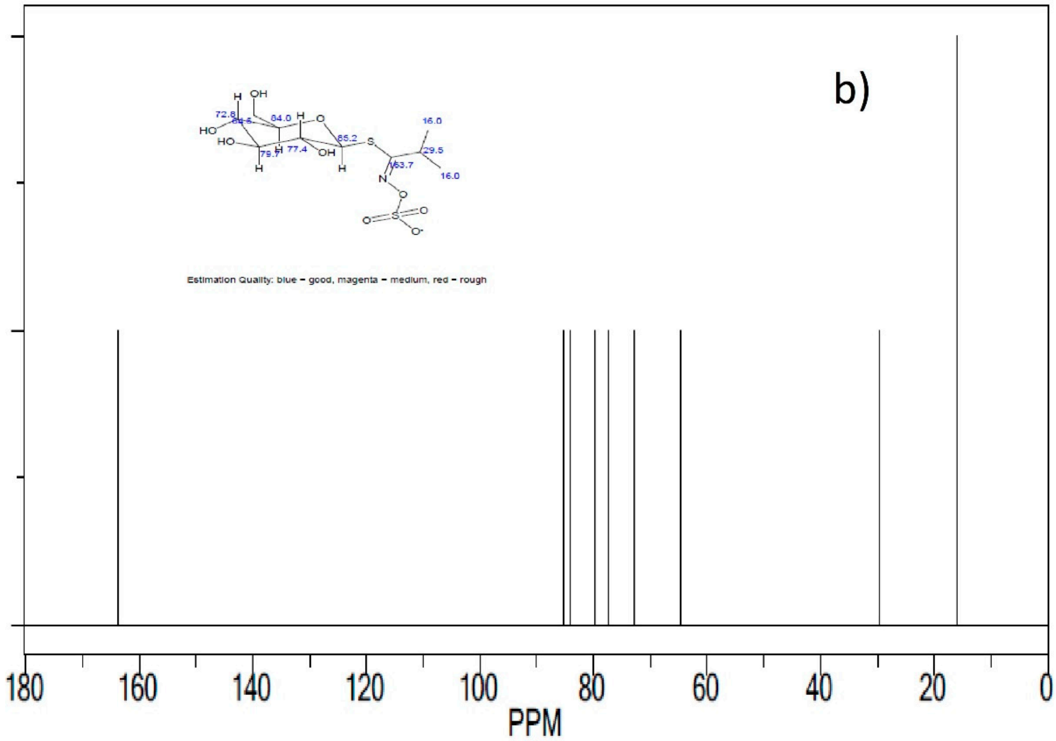

Figure S8: High Resolution mass spectra of compound **1**.

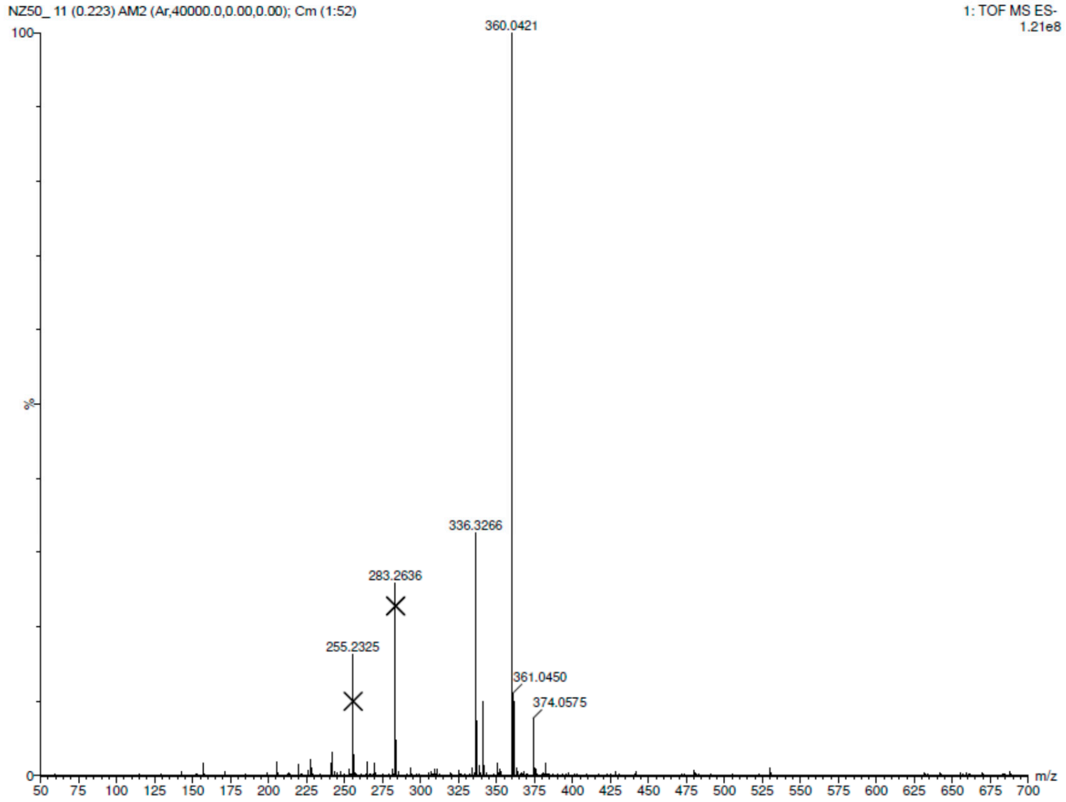

Elemental Composition Report

Page 1

Single Mass Analysis

Tolerance = 5.0 PPM / DBE: min = -1.5, max = 200.0
Element prediction: Off
Number of isotope peaks used for i-FIT = 5

Monoisotopic Mass, Even Electron Ions
210 formula(e) evaluated with 1 results within limits (all results (up to 1000) for each mass)
Elements Used:
C: 5-30 H: 10-100 N: 1-1 O: 0-10 Na: 0-1 S: 0-2

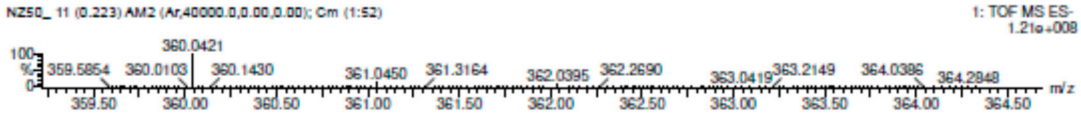

Minimum:				-1.5				
Maximum:		5.0	5.0	200.0				
Mass	Calc. Mass	mDa	PPM	DBE	i-FIT	Norm	Conf(%)	Formula
360.0421	360.0423	-0.2	-0.6	2.5	2727.7	n/a	n/a	C10 H18 N O9 S2

Figure S9: Infrared spectrum of compound **1**.

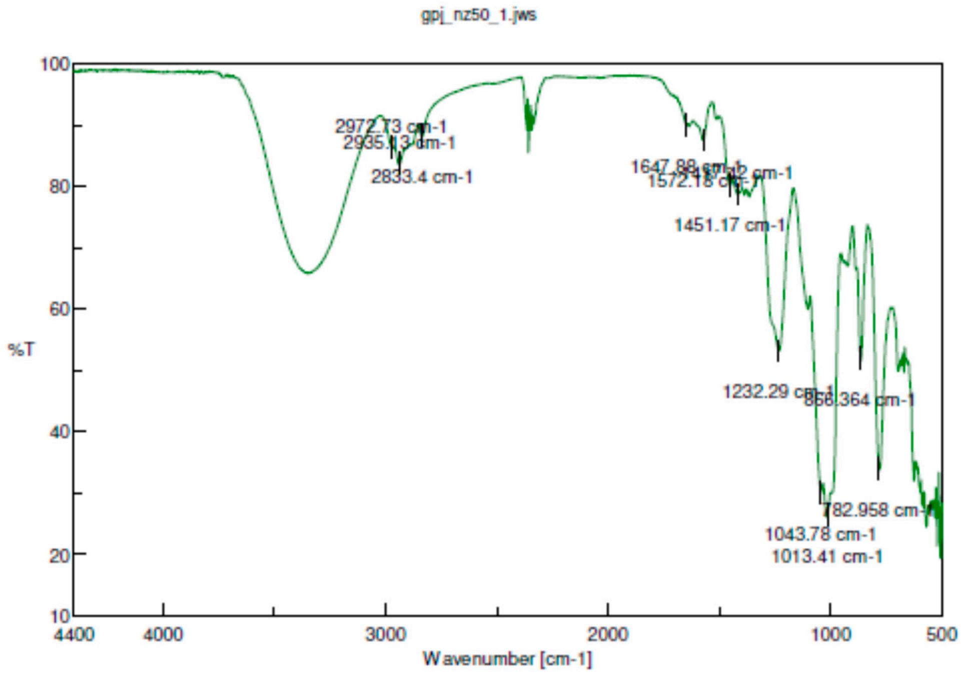

gpj_nz50_1.jws

[Comments]	
Sample name	
Comment	
User	
Division	
Company	UNIMI

[Measurement Information]	
Model Name	FT/IR-4600typeA
Serial Number	D063961786
Accessory	ATR-PRO-ONE
Accessory S/N	B109661809
Incident angle	45 deg
Measurement Date	2/20/2019 10:57 AM
Light Source	Standard
Detector	TGS
Accumulation	32
Resolution	2 cm-1
Zero Filling	On
Apodization	Cosine
Gain	Auto (8)
Aperture	Auto (5 mm)
Scanning Speed	Auto (2 mm/sec)
Filter	Auto (30000 Hz)

[Detailed Information]	
Creation date	2/20/2019 11:05 AM
Date modified	2/20/2019 2:38 PM
Data array type	Linear data array
Horizontal axis	Wavenumber [cm-1]
Vertical axis	%T
Start	499.955 cm-1
End	4400.28 cm-1
Data interval	0.482117 cm-1
Data points	8091

Figure S10: TRPA1 is activated by IPITC (a), 2-BITC (b) and AITC (c, for comparison). The graphs show the representative traces of [Ca2+]i increase evoked by the three agonists at 100 μM in HEK293 cells over-expressing rat TRPA1. For IPITC (a) and 2-BITC (b) the subsequent desensitization of the AITC (100 μM) effect is also shown.

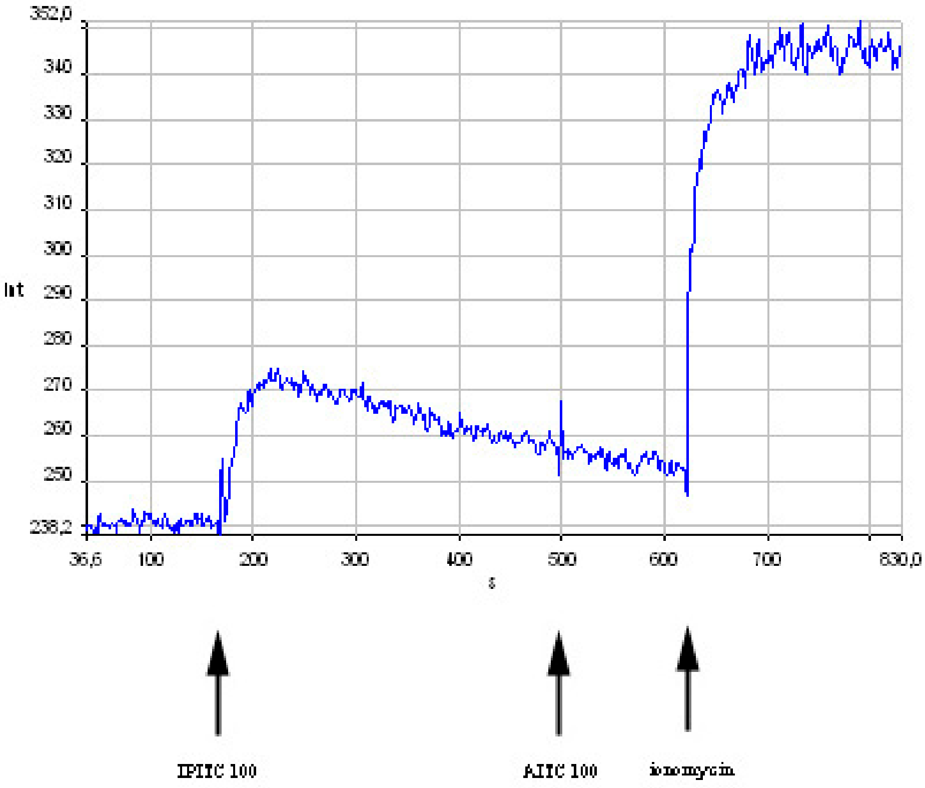

Figure S10 a). IPITC =isopropylisothiocyanate, compound **2**

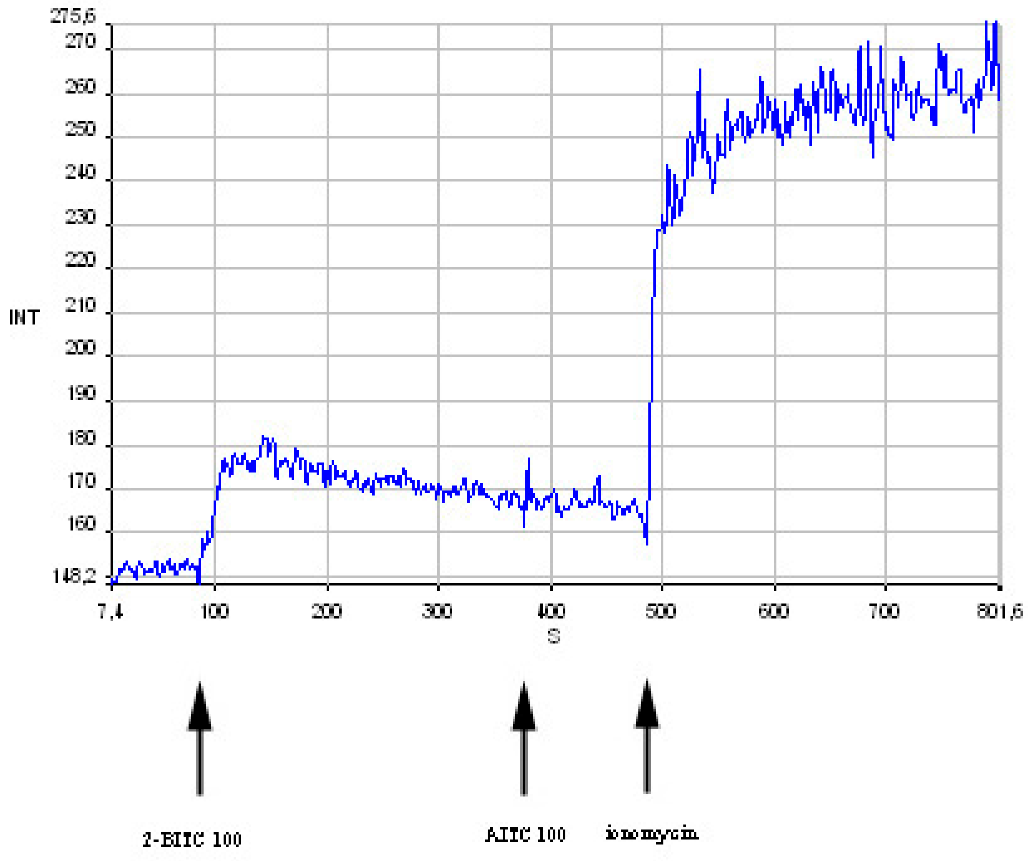

Figure S10, b). 2-BITC = 2-butylisothiocyanate, compound **4**

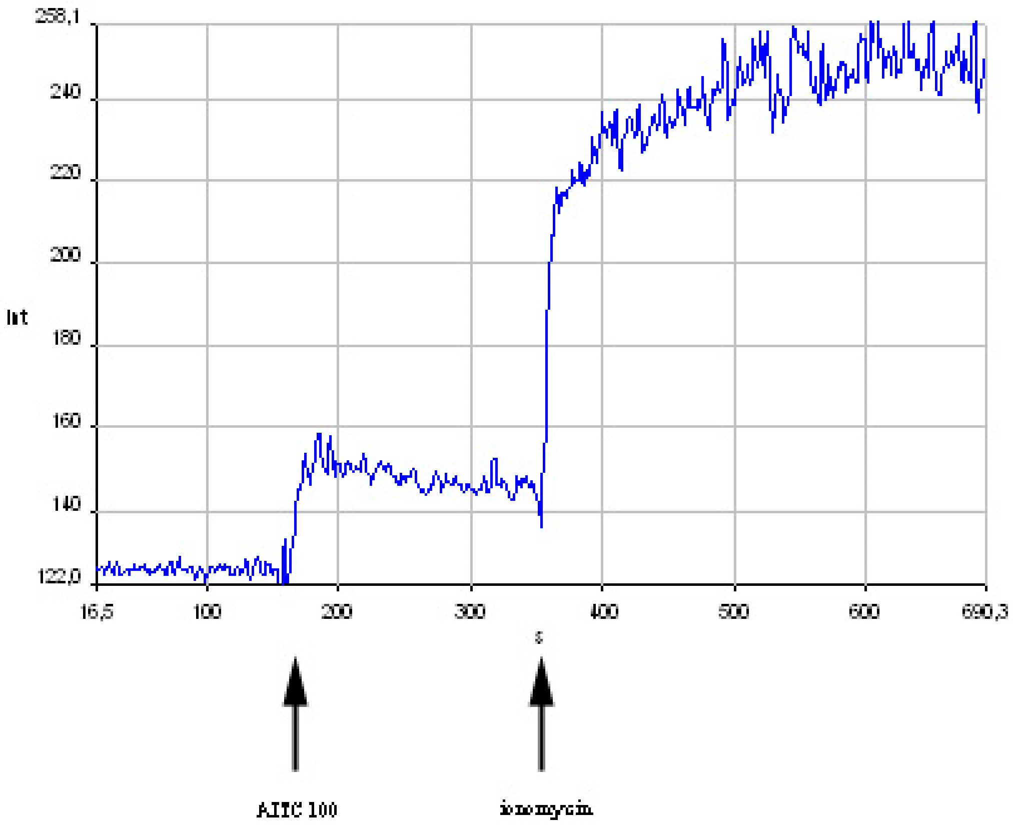

Figure S10, c) AITC=allylisothiocyanate, compound **6**
